# Phosphoinositide-3 Kinase-Akt Pathway Controls Cellular Entry of Ebola Virus

**DOI:** 10.1371/journal.ppat.1000141

**Published:** 2008-08-29

**Authors:** Mohammad F. Saeed, Andrey A. Kolokoltsov, Alexander N. Freiberg, Michael R. Holbrook, Robert A. Davey

**Affiliations:** 1 Department of Microbiology and Immunology, University of Texas Medical Branch, Galveston, Texas, United States of America; 2 Western Regional Center of Excellence in Biodefense and Emerging Infectious Diseases Research, University of Texas Medical Branch, Galveston, Texas, United States of America; 3 Department of Pathology, University of Texas Medical Branch, Galveston, Texas, United States of America; 4 Institute of Human Infection and Immunity, University of Texas Medical Branch, Galveston, Texas, United States of America; Harvard Medical School, United States of America

## Abstract

The phosphoinositide-3 kinase (PI3K) pathway regulates diverse cellular activities related to cell growth, migration, survival, and vesicular trafficking. It is known that Ebola virus requires endocytosis to establish an infection. However, the cellular signals that mediate this uptake were unknown for Ebola virus as well as many other viruses. Here, the involvement of PI3K in Ebola virus entry was studied. A novel and critical role of the PI3K signaling pathway was demonstrated in cell entry of Zaire Ebola virus (ZEBOV). Inhibitors of PI3K and Akt significantly reduced infection by ZEBOV at an early step during the replication cycle. Furthermore, phosphorylation of Akt-1 was induced shortly after exposure of cells to radiation-inactivated ZEBOV, indicating that the virus actively induces the PI3K pathway and that replication was not required for this induction. Subsequent use of pseudotyped Ebola virus and/or Ebola virus-like particles, in a novel virus entry assay, provided evidence that activity of PI3K/Akt is required at the virus entry step. Class 1A PI3Ks appear to play a predominant role in regulating ZEBOV entry, and Rac1 is a key downstream effector in this regulatory cascade. Confocal imaging of fluorescently labeled ZEBOV indicated that inhibition of PI3K, Akt, or Rac1 disrupted normal uptake of virus particles into cells and resulted in aberrant accumulation of virus into a cytosolic compartment that was non-permissive for membrane fusion. We conclude that PI3K-mediated signaling plays an important role in regulating vesicular trafficking of ZEBOV necessary for cell entry. Disruption of this signaling leads to inappropriate trafficking within the cell and a block in steps leading to membrane fusion. These findings extend our current understanding of Ebola virus entry mechanism and may help in devising useful new strategies for treatment of Ebola virus infection.

## Author Summary

Each year, filoviruses such as Ebola virus claim many human lives and decimate gorilla populations in Africa. Infection results in an acute fever often associated with profuse internal and external bleeding and death rates of up to 90%. Due to these symptoms and high pathogenicity, these viruses have been heavily publicized in the media. The first step of infection is entry, where the virus is taken up and penetrates into the cell, from which it spreads throughout the body. While it is known that the cell must engulf the virus by the process of endocytosis, we know little about how the virus triggers this event. Here, we use a novel technology to measure penetration of Ebola virus into the cell in real time and show that Ebola virus stimulates phosphoinositide-3 kinase, a signaling molecule known to induce endocytosis. Importantly, drugs that interfere with this signaling inhibit infection by Ebola virus and block virus spread. This work provides a mechanistic insight into how Ebola virus manipulates the cell to start an infection, may explain part of virus induced pathogenesis, and provides a potential way to treat this deadly disease.

## Introduction

Ebola virus, a member of the family *Filoviridae*, is an emerging infectious agent that causes severe and often fatal hemorrhagic fever in humans and nonhuman primates. In many outbreaks, especially those caused by Zaire Ebola virus (ZEBOV), mortality rates close to 90% have been reported [1]. Currently, no vaccine or therapy is available for Ebola virus hemorrhagic fever. Significant mortality rate, high transmissibility and lack of therapeutic and preventive measures make Ebola virus a potentially serious public health threat.

Ebola viruses are filamentous enveloped viruses. The envelope contains two virally-encoded glycoproteins, GP1 and GP2, which together serve as the primary viral determinant for entry into host cells. The two glycoproteins are produced from a precursor protein (GP), which is cleaved by a furin-like endoprotease to generate surface-bound protein GP1 and the transmembrane protein GP2, the two proteins, remain associated by a disulfide bridge after cleavage [2]. As with many enveloped viruses, entry of ZEBOV into cells likely involves virus particles binding to host cell receptor(s), followed by endocytosis and trafficking through vesicular compartments, and finally fusion of the virus membrane to that of the endocytic vesicle. This results in release of the viral nucleocapsid into the cytoplasm where the subsequent steps of the replication cycle take place [3]. GP1 is believed to mediate interaction with the host cell receptor, while GP2 is involved in membrane fusion [3]. Membrane fusion is believed to involve a GP2 structural rearrangement triggered by low pH in an endocytic compartment. In addition, intracellular processing of GP1 by endosomal cathepsins is also a prerequisite to membrane fusion [4]. Thus, an important aspect of ZEBOV entry involves endocytic trafficking into the cell.

Recent work has shown that many viruses exploit host cell molecules and signaling pathways to facilitate various steps of the entry process. A critical role of focal adhesion kinase and protein kinase C was described for endocytosis and endosomal sorting of West Nile virus in mosquito cells [5], and recently, Rho A and its upstream tyrosine kinases were implicated in endocytosis and trafficking of poliovirus [6]. However, for most viruses, especially enveloped viruses, information on the requirements of cell signaling for entry is limited. Important advances have been made regarding the entry of Ebola virus and the role of the envelope glycoproteins in cell attachment and endocytosis (reviewed in [7]). However, our understanding of the role of cell signaling in virus entry remains limited.

The phosphoinositide-3 kinase (PI3K) pathway is an important cell signaling pathway that regulates diverse cellular activities including proliferation, differentiation, apoptosis, migration, metabolism, and vesicular trafficking [8]. PI3Ks (OMIM#601232; Online Mendelian Inheritance in Man database: http://www.ncbi.nlm.nih.gov/sites/entrezdbomim) are a family of lipid kinases that are divided into three classes according to their structure and substrate specificity. Of these, class I PI3Ks are the most widely studied. They signal through cell surface protein tyrosine kinase (PTK) or G-protein coupled (GPC) receptors. Activation of PI3K results in phosphorylation of phosphatidylinositol-bis phosphates to produce phosphatidylinositol-triphosphates, which serve as potent second messengers for downstream signaling. Akt-1 (OMIM#164730), is a key downstream intermediate in PI3K-dependent signaling. A variety of molecules are directly or indirectly regulated by Akt, and serve as downstream effectors to carry out diverse PI3K-regulated responses [9].

Regulation of vesicular trafficking is one of the oldest recognized functions of PI3Ks [10]. PI3Ks influence a variety of intracellular trafficking events that include cargo selection, vesicle formation, vesicle movement and membrane fusion. This is often through stimulation of actin turnover [11]. Rac1 (OMIM#602048), along with other Rho family GTPases, is a key effector in this process [12]. Therefore the regulation of Rac1 by class 1 PI3Ks, as seen in many cell types [13]–[16], provides a mechanism to couple receptor-ligand interaction to induction of endocytosis as well as other actin-mediated functions in the cell. Bacterial pathogens have been shown to take advantage of this mechanism by stimulating phagocytosis and internalization through PI3K activation [17],[18]. Similarly, interaction of the non-enveloped adenovirus with receptors on the cell surface was shown to activate Rac1 and Cdc42 (OMIM#116952) in a PI3K-dependent manner, and this was required for virus uptake into endosomes [19]. However, similar dependencies for enveloped viruses have not been described. Here we investigated the role of PI3K cell signaling pathway in cellular entry of ZEBOV. The findings indicated that ZEBOV induces activation of PI3K pathway prior to or during entry and that activity of class 1A PI3Ks is critical for entry into host cells. Rac1 GTPase was found to be an important downstream effector in regulating ZEBOV entry. The impact of inhibiting PI3K, Akt or Rac1 was similar, causing an aberrant accumulation of ZEBOV particles in intracellular vesicles, indicating a role of the PI3K-Akt-Rac1 pathway in vesicular trafficking of virus particles.

## Results

### PI3K activity is important for an early event(s) in ZEBOV infection

**Figure 1:**
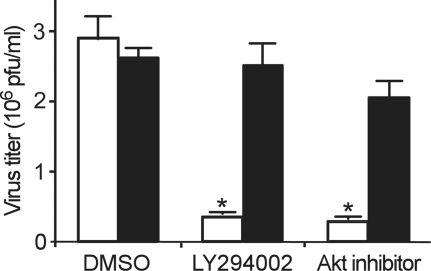
PI3K is important for early step(s) during ZEBOV infection. Vero-E6 cells were incubated with LY294002 (50 µM) or Akt inhibitor (10 µM) for 2 h in the presence of virus. Unbound virus and drug was then removed and cells were cultivated. After 10 d, cells were fixed in formalin and stained with crystal violet; plaques were then counted. Effect of the drugs on VSV (Indiana strain) infection was also tested similarly, except that plaques were counted 2 d after inoculation of cells. Open bars, ZEBOV; solid bars, VSV (* *p*<0.01).

Several viruses utilize the PI3K-Akt pathway to support replication in host cells [20]–[23], however, involvement of this pathway in early events of infection such as entry has not been conclusively demonstrated for enveloped viruses. We investigated the effect of LY294002 (a highly specific inhibitor of PI3K) on infection by wild type ZEBOV. To determine if the drug was acting early or late in the infection cycle, cells were exposed to drug only during the first 2 h of incubation with the virus. Subsequently, the drug and the unbound virus were removed, and infection was allowed to continue. As compared to untreated cells, the infection of ZEBOV was significantly (nearly 10-fold) reduced in cells treated with LY294002 (*p*<0.01). In contrast, LY294002 exhibited no significant effect (*p*>0.05) on infection by vesicular stomatitis virus (VSV). A similar level of inhibition of ZEBOV infection was observed when a specific Akt inhibitor was used (Figure 1). These data suggested that the PI3K-Akt pathway plays a role in one or more of the early events in the ZEBOV infection cycle.

### ZEBOV interaction with cells directly stimulates activation of the PI3K pathway

**Figure 2:**
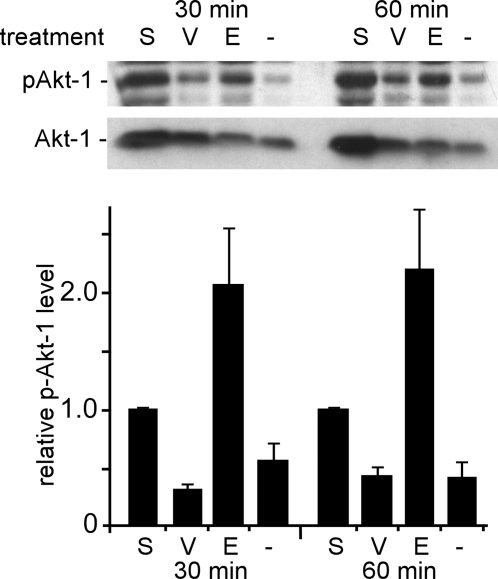
ZEBOV particles induce Akt-1 phosphorylation early during infection. Serum-starved HEK293 cells were incubated with serum-free medium (−), medium containing 10% FBS (S), and serum-free medium containing γ-radiation–inactivated ZEBOV (E, MOI = 5) or VSV (V, MOI = 5) at 37°C for 30 or 60 min. Subsequently, cells were washed and lysed, and phosphorylated Akt-1 was detected by Western blotting (p-Akt-1; top panel). The same membrane was then stripped and re-probed for total Akt-1 (bottom panel). The bar graph shows a summary of densitometry analysis of bands, which was performed using the ImageJ gel analysis package. The ratio of band intensities of phospho-Akt-1 to total Akt-1 was determined and normalized to the serum-treated control. Each data point represents mean±SD of 3 experiments.

While the above data indicated that the activity of PI3K is important for early event(s) in ZEBOV replication, it was unclear if the basal level activity of the PI3K-Akt pathway was sufficient for infection or if ZEBOV itself was capable of inducing this pathway to promote infection. To address this question, phosphorylation of Akt-1 was measured in cells after incubation with ZEBOV. Akt is a major downstream effector of the PI3K pathway and is phosphorylated (activated) following activation of PI3K. Therefore, Akt phosphorylation is often used as an indirect, but reliable measure of PI3K pathway activation [21]–[23]. Serum-starved HEK293 cells were incubated with medium containing no serum (negative control), medium containing 10% fetal bovine serum (positive control), or γ-radiation-inactivated ZEBOV or VSV. Compared to the negative control, ZEBOV caused a marked (>2-fold) increase in Akt phosphorylation within 30 min and actually surpassed the level observed after serum stimulation of cells (Figure 2). In contrast, VSV had no significant effect on phosphorylation of Akt-1 over this time interval. These data suggested that ZEBOV actively and strongly induces the PI3K pathway very early during the infection process. Since γ-radiation-inactivated virus had been used, it was likely that this stimulation was the product of direct GP interaction with cell receptors.

The early dependence of ZEBOV infection on PI3K and Akt activity, and a significant induction of Akt phosphorylation by γ-radiation-inactivated (replication-incompetent) ZEBOV suggested that the PI3K pathway is likely involved at an early step in infection, most likely entry.

### The PI3K-Akt pathway is critical for entry of ZEBOV

To investigate if the PI3K-Akt pathway played a role in ZEBOV entry or was required for some other early, but post-entry step, we adapted a previously described contents mixing assay that allowed rapid and quantitative measurement of entry of diverse enveloped viruses up to and including the point of membrane fusion [24]. The assay, which was originally based on virus pseudotypes, measures release of a recombinant nef-luciferase protein, encapsulated within virus particles. The nef peptide serves to non-specifically target luciferase to cell membranes at the time of particle budding and so, incorporates luciferase within the membrane of new virus particles. After cell-virus membrane fusion, the luciferase becomes accessible to its substrates, previously loaded into cells, and light is emitted. Here the assay was adapted for use, first with ZEBOV GP pseudotyped particles and then with ZEBOV virus-like particles. Both types of virus particles give a measure of GP function but VLPs, which share a filamentous structure with native ZEBOV are likely a better model system for wild type virus. In either case, this is the first time that this assay technology has been adapted to ZEBOV. Each assay was applied to determine if the PI3K-Akt pathway played a role in entry of ZEBOV.

To assess their efficiency in an entry assay, luciferase-containing pseudotypes with ZEBOV envelope glycoproteins (EVP) or VSV-G protein (VSVP), and VLPs carrying ZEBOV envelope glycoproteins (ZEBO-VLP) or VSV-G protein (VSV-VLP) were produced, sucrose purified and tested on cells. As compared to particles devoid of envelope glycoproteins, a strong signal was obtained for each of EVP, VSVP, ZEBO-VLP and VSV-VLP (Table 1, column 2). The relatively large difference in the signal between EVP and VSVP correlated to differences in pseudotype virus titer, as determined by standard infection assays with a GFP reporter gene (Table 1, column 3) and likely reflects the potency of the VSV-G relative to ZEBOV GP.

**Table 1:** Activity of luciferase-containing pseudotyped virus and virus-like particles in the entry assay.

	Luciferase Activity (counts/µl of Virus)^a^	Virus Titer (cfu/ml)^b^
Pseudotype with no envelope protein	0.7±0.2	1.7±0.7×10^2^
EVP	150±50	1.2±0.5×10^5^
VSVP	3,000±500	2.5±1.5×10^7^
VLP with no envelope protein	5±3	ND
ZEBO-VLP	673±171	ND
VSV-VLP	1,272±57	ND

ND, not determined; VLPs do not encode an expressed transgene to allow titer determination.

a Pseudotyped virus particles or VLPs were pelleted through sucrose and resuspended in 0.01 volumes of the original culture volume. A total of 0.2 ml of virus was used per assay with 10^6^ cells and values were measured after 3 h of incubation.

b Virus was prepared as above and used to infect HEK293-mCAT-1 cells. Titer was determined by limiting dilution using GFP reporter expression to count infected cells. Values for titer and entry assay are the mean±SD of 3 independent experiments.

**Figure 3:**
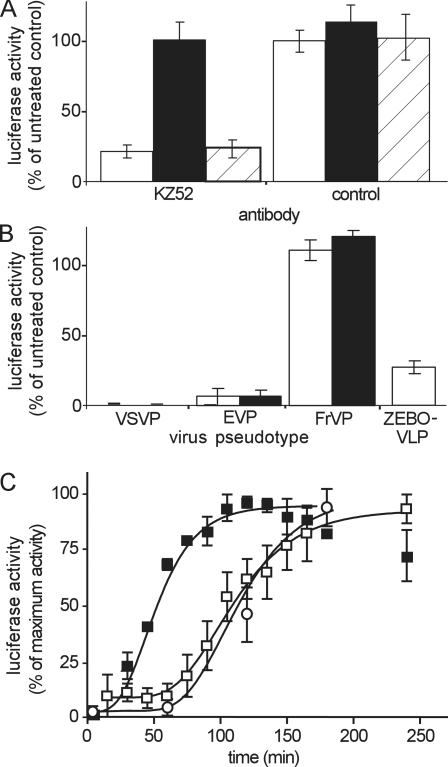
Validation of luciferase virus entry assay for ZEBOV pseudotypes and VLPs. (A) The luciferase entry assay was performed using EVP (open bars) or VSVP (solid bars) or ZEBO-VLP (diagonally hatched bars) on HEK293-mCAT-1 cells in the presence of anti-Ebola virus neutralizing antibody (KZ52, 0.3 µg/ml) or a control antibody of irrelevant specificity (anti-HA, 12CA5; Roche, 0.3 µg/ml). Data were normalized to luciferase activity in cells incubated with untreated virus. Each data point represents the mean±SD of 3 independent experiments. (B) Luciferase entry assays were performed using VSVP, EVP, or FrVP on HEK293-mCAT-1 cells in the presence of either 20 mM ammonium chloride (open bars) or 50 nM bafilomycin A1 (solid bars). Effect of ammonium chloride was also tested on ZEBO-VLP. Data were normalized to luciferase activity for untreated cells. Each data point represents the mean±SD of 3 independent experiments. (C) Kinetics of entry was measured for EVP (open squares), VSVP (closed squares), and ZEBO-VLP (open circles). Each was incubated with HEK293-mCAT1 cells at room temperature. After 10 min, cells were washed to remove unbound virus and incubated at 37°C. At indicated time intervals, aliquots of cells were withdrawn, and luciferase signals were measured. For each virus, luciferase activity at different time points was normalized to the maximum luciferase activity for that virus. Each data point represents the mean±SD of 2 independent experiments.

Both EVP and ZEBO-VLPs were then further validated for specificity of entry into cells. In each case the activity of the pseudotyped particles reflected that of the VLPs. ZEBOV neutralizing antibody (KZ52) significantly (∼70%) blocked entry of both EVP and ZEBO-VLP, (Figure 3A) and correlated to that reported for inhibition of infectious ZEBOV infection at the concentration of the antibody used here [25] while entry of VSVP was unaffected. Secondly, ammonium chloride (NH4Cl) and bafilomycin A1, two well-known inhibitors of endosomal acidification, both inhibited the pH-dependent entry of EVP and VSVP, while they had no effect on entry of a Friend murine leukemia virus pseudotype (FrVP), a pH-independent virus. Similarly, entry of ZEBO-VLP was also inhibited by NH_4_Cl (Figure 3B). Thirdly, a detailed examination of the entry kinetics of EVPs and ZEBO-VLPs (Figure 3C) revealed that the peak of the entry signal was preceded by a pronounced lag and occurred much later than that for VSV-G bearing particles. This timing was similar to that reported previously for a pseudotype infection assay [26]. Of note, both pseudotypes and VLPs entered cells with similar behavior and timings, indicating that the GP dictated the uptake kinetics and pathway used by the virus particle, more than the particle shape. In subsequent studies, the majority of the presented data are from VLPs but similar outcomes were seen with pseudotyped particles and are shown for comparison.

**Figure 4:**
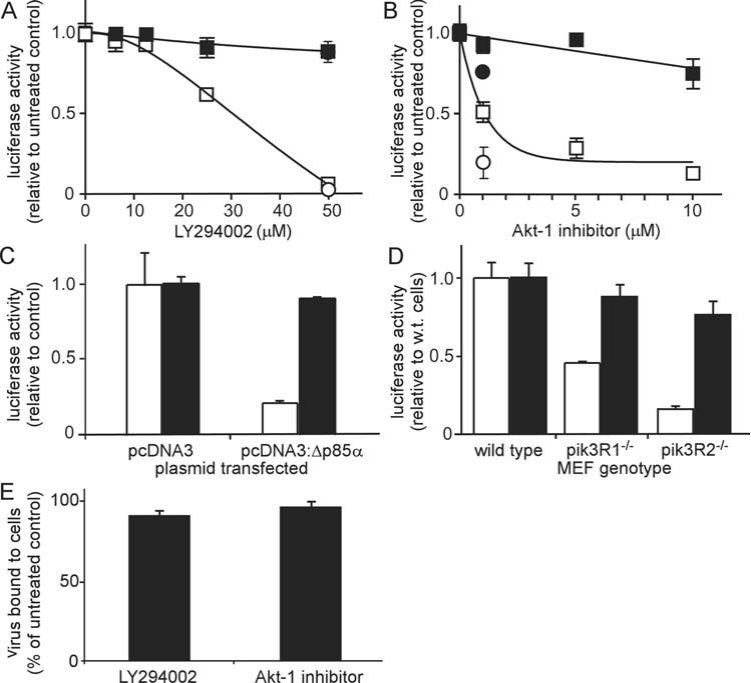
PI3K-Akt-1 pathway plays a critical role in Ebola virus entry. (A) HEK293-mCAT-1 cells were pre-treated with the indicated concentrations of LY294002 for 1 h followed by incubation with ZEBO-VLP (open squares), VSV-VLP (closed squares), EVP (open circle), or VSVP (closed circle) for 3 h in the continued presence of the drug. Entry signals were then measured. Data were normalized to luciferase activity in DMSO (vehicle)-treated cells. (B) HEK293-mCAT-1 cells were pre-treated with the indicated concentrations of Akt inhibitor for 1 h followed by incubation with ZEBO-VLP (open squares), VSV-VLP (closed squares), EVP (open circle), or VSVP (closed circle) for 3 h in the continued presence of the drug. Entry signals were then measured. Data were normalized to luciferase activity in DMSO (vehicle)-treated cells. (C) HEK293-mCAT-1 cells were transfected with either pcDNA3 or pcDNA3 encoding a dominant-negative form of the PI3K p85-α regulatory subunit (Δp85α). Entry assays were performed 36 h after transfection using ZEBO-VLP (open bars) or VSV-VLP (solid bars). Data were normalized to luciferase activity in untransfected cells. Each data point represents the mean±SD of 3 experiments. (D) Wild-type (WT), pik3R1^−/−^ (p85α, p55α, and p50α-deficient), or pik3R2^−/−^ (p85β -deficient) mouse embryonic fibroblast (MEF) cells were incubated with EVP (open bars) or FrVP (solid bars). After 3 h, cells were washed, and luciferase activity was measured. Each data point represents the mean±SD of 3 independent experiments. (E) HEK293-mCAT-1 cells were pre-treated with LY294002 (50 µM) or Akt-1 inhibitor (1.0 µM) for 1 h at 37°C, followed by incubation with ZEBO-VLPs for 10 min at room temperature. Cells were then washed to remove unbound virus and resuspended in luciferase assay buffer containing triton X-100 detergent; luciferase activity was then measured. Data were normalized to luciferase activity in vehicle-treated samples. Each data point represents the mean±SD of 2 experiments.

To investigate if the PI3K-Akt pathway played a role during the entry steps of ZEBOV infection, LY294002 and Akt inhibitor were tested in the entry assay. In each case, cells treated with the inhibitors exhibited significant reduction in the entry of ZEBOV GP bearing particles but not for particles bearing VSV-G (Figs. 4A, B). As a further independent test, a dominant-negative mutant of the p85 regulatory subunit (OMIM#171833) of class 1A PI3K (Δp85α) was used to inhibit PI3K-mediated signaling. Δp85α retains the ability to bind phosphotyrosine residues on upstream receptors that signal through PI3K but lacks the ability to interact with the PI3K catalytic domain [27]. Cells were transiently transfected with either a control plasmid (pcDNA3) or plasmid expressing Δp85α (pcDNA3:Δp85α) and entry assays performed using ZEBO-VLPs or VSV-VLPs (Figure 4C). Entry of ZEBO-VLPs was significantly inhibited in cells expressing Δp85α as compared to that in cells transfected with the empty vector, indicating a role for this isoform. In contrast, the entry of VSV-G bearing particles was similar in both cell types (Figure 4C).

Δp85α inhibits activity of those PI3K heterodimers that contain the α-isoform of the p85 regulatory subunit. To test the potential involvement of other isoforms, entry assays were performed using pik3R1 (OMIM#171833) knockout (p85α-, p50α-, p55α-deficient) or pik3R2 (OMIM#603157) knockout (p85β-deficient) mouse embryonic fibroblasts (MEF). Due to lower sensitivity of the VLP-based assay system in these cells, the experiments were performed using the pseudotyped virus-based assay. Furthermore, because of intrinsic resistance of corresponding wild-type MEFs to infection by VSVP, FrVPs were again used as a control. Compared to wild-type MEFs, EVP entry was reduced by >50% in the homozygous knockout cells for pik3R1, while entry in pik3R2 knockout cells was reduced by 75%. No significant effect on the entry signal of the FrVP was observed in either cell type (Figure 4D). These data indicate that both p85α and p85β containing PI3K heterodimers are involved in entry of ZEBOV.

The above findings strongly indicated that inhibition of PI3K-Akt pathway blocked ZEBOV infection up to or including the membrane fusion step. However, this could be due to impaired virus binding to cells or inhibition at a post-binding step such as endocytosis, trafficking, or membrane fusion. To further define the mechanism by which PI3K controls ZEBOV entry, virus binding to cells was measured. Cells were pretreated with LY294002 or Akt-1 inhibitor and pseudotyped viruses were bound for 10 min. Unbound virus was then washed away and cells with residual bound particles were lysed using non-ionic detergent to release virus encapsulated luciferase. Compared to DMSO-treated (control) cells, no significant difference was observed in luciferase activity in samples that were treated with either LY294002 or Akt-1 inhibitor, indicating that ZEBOV pseudotype binding to cells was unaffected (Figure 4E). Similarly, no significant difference was observed among p85 wild-type, pik3R1^−/−^ or pik3R2^−/−^MEFs in their capacity to bind the pseudotyped particles (data not shown). This suggested that inhibition of the PI3K-Akt pathway does not influence levels of accessible receptor on the cell surface. Therefore, the PI3K-Akt pathway most likely plays a role in one or more post-binding steps involved in entry.

### Rac1 serves as a downstream effector of the PI3K-Akt pathway in ZEBOV entry

Among the possible downstream effectors regulated by PI3K-Akt pathway, mTOR (OMIM#601231) and Rac1 are most likely to influence the entry process. mTOR, being a positive regulator of translation [28], could potentially stimulate synthesis of factors needed during entry. However, a role of mTOR in ZEBOV entry was ruled out as rapamycin, a potent inhibitor of mTOR, had no effect on EVP entry (data not shown).

**Figure 5:**
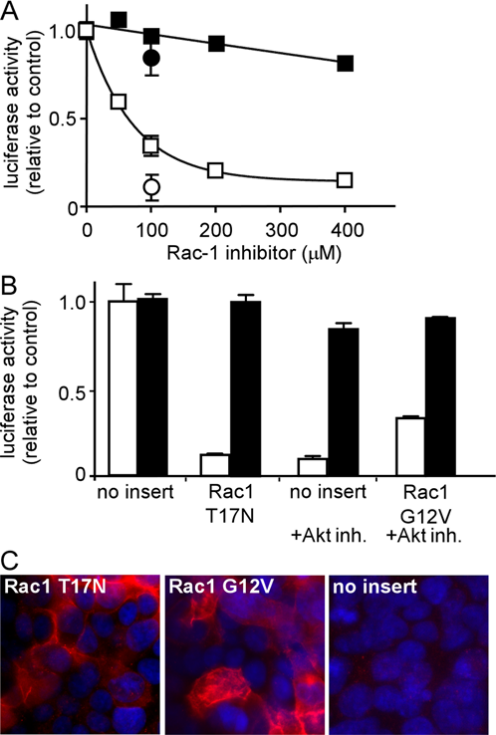
Rac1 plays a role in ZEBOV entry and serves as a downstream effector of Akt-1 signaling. (A) HEK293-mCAT-1 cells were treated with indicated concentrations of Rac1 inhibitor, and entry assays were performed using ZEBO-VLP (open squares), VSV-VLP (closed squares), EVP (open circles), or VSVP (closed circles). Data points represent the average±SD of 2 experiments. (B) HEK293-mCAT-1 cells were transfected with either pcDNA3 vector lacking insert, or pcDNA3 encoding dominant-negative Rac1 (Rac1-T17N) or pcDNA3 encoding constitutively active Rac1 (Rac1-G12V). Entry assays were performed 36 h later using ZEBO-VLPs (open bars) or VSV-VLP (solid bars) in the absence or presence of Akt inhibitor. Results are expressed as the percent of luciferase activity in untransfected cells. Each data point represents the mean±SD of 2 experiments. (C) Cells transfected with plasmids encoding HA-tagged Rac1-T17N (left panel), Rac1-G12V (middle panel), or empty vector (right panel) were stained using anti-HA mouse monoclonal antibody and Alexafluor_594_-conjugated anti-mouse IgG.

Rac1, through its regulation of actin polymerization plays a vital role in various steps involved in endocytosis. In many cell types, activity of Rac1 is regulated by the PI3K-Akt pathway [13]–[16]. Therefore, a specific Rac1 inhibitor was tested. A dose dependent inhibition of ZEBO-VLP was observed peaking at >90% inhibition at 400 µM without affecting VSV-VLP entry (Figure 5A). Furthermore, transient expression of a dominant-negative Rac1 mutant (Rac1-T17N) in cells reduced entry by >80% (Figure 5B). The extent of ZEBO-VLP entry inhibition corresponded to the number of cells expressing the dominant-negative Rac1 (Figure 5C, left panel).

The above findings provided evidence that PI3K-Akt and Rac1 pathways play a role in ZEBOV entry; however, it remained unclear if the two pathways were linked or acted independently. To investigate this, cells were made to express a constitutively active form of Rac1 (Rac1-G12V) in the presence of the Akt inhibitor. The effects of the inhibitor were significantly reversed (Figure 5B) by the mutant, indicating, that PI3K-Akt and Rac1 act sequentially in a pathway that controls entry of ZEBOV, with PI3K-Akt acting upstream of Rac1. Again, the extent of the effect of constitutively active Rac1 was proportional to the number of cells expressing the mutant protein (Figure 5C, middle panel). Similar data were obtained when EVPs and VSVPs were used in the assay (data not shown).

### Inhibition of PI3K-Akt-Rac1 pathway leads to a blockade in ZEBOV uptake

**Figure 6:**
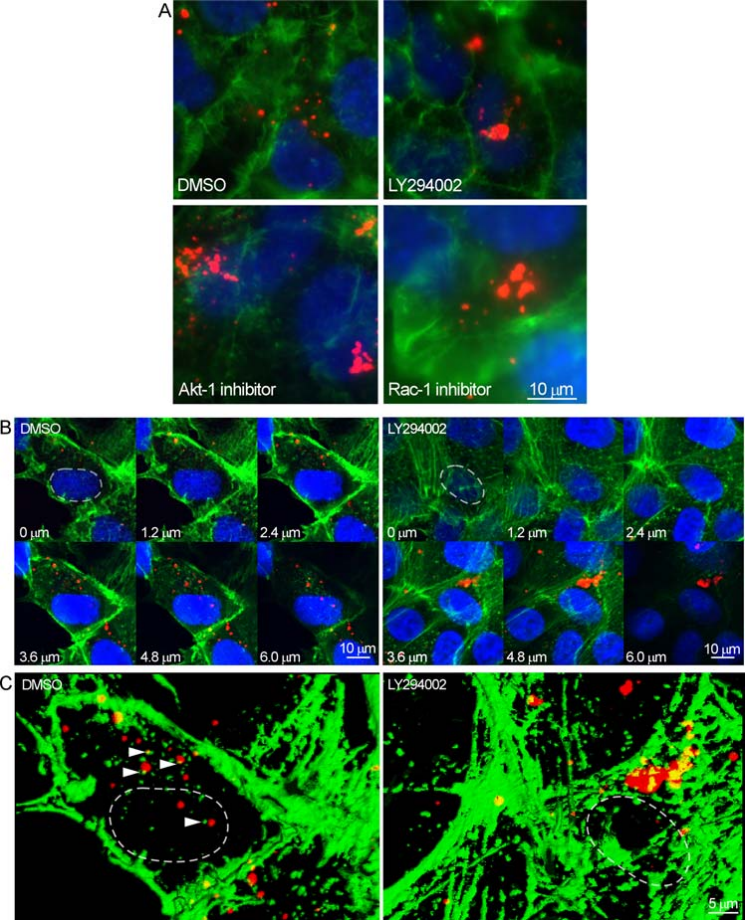
Inhibitors of PI3K, Akt, and Rac1 cause intracellular clustering of ZEBOV particles. (A) HEK293-mCAT-1 cells were grown on chambered coverglass and treated with DMSO (control) or the indicated drugs for 1 h, followed by incubation with fluorescently labeled ZEBOV (red) for 3 h. Cells were fixed, permeabilized, and incubated with Alexafluor488-labeled phalloidin to visualize F-actin and DAPI to stain nuclei (blue). Subsequently, slides were washed and photographed by fluorescence microscopy using a 100× oil immersion objective. Scale bars are shown at lower right and apply to all images in the panel. (B) The observations made using HEK293-mCAT-1 cells were confirmed in Vero-E6 cells by confocal microscopy. (Top panel) Serial optical sections of cells treated for 1 h with DMSO (left) or the PI3K inhibitor LY294002 (right) and then incubated with fluorescently labeled ZEBOV (red) for 2 h. Cells were fixed and stained with phalloidin to label F-actin (green) and DAPI to label cell nuclei (blue). The level of the section is indicated, with 0 on the basolateral cell surface and 6 µm close to the apical surface of the cell. (C) Volume projection of all optical z-sections (computed using ImageJ software) of representative cells incubated in the presence of DMSO or LY294002. In DMSO-treated cells, examples of actin bundles adjacent to virus particles are indicated by arrowheads. Only the red and green fluorescence channels are shown, and the nuclei of chosen cells are indicated with dashed lines.

Inhibitors of PI3K, Akt and Rac1 had no significant effect on EVP binding to cells (Figure 4E, and data not shown) indicating that the action of the PI3K-Akt-Rac1 pathway was likely important for endocytosis, trafficking and/or membrane fusion of ZEBOV. To examine this, ZEBOV particles were labeled with a red fluorescent dye (Alexa Fluor_594_) and incubated with HEK293 cells in the absence or presence of LY294002, Akt inhibitor or Rac1 inhibitor. Cells were also stained for F-actin to visualize the cell cytoskeleton, and analyzed by confocal microscopy. In untreated cells, particles were found distributed evenly throughout the cell cytoplasm. In contrast, after treatment with each inhibitor, particles accumulated in clusters (Figure 6A). The drugs were also tested with Vero-E6 cells, a widely used, ZEBOV permissive cell line. Again, in untreated cells, individual virus particles were distributed throughout the cell cytoplasm (Figure 6B). Interestingly, image analysis of serial z-sections revealed that most of the individual particles were adjacent to small actin bundles (Figure 6C, left panel, arrow heads) supporting a requirement for actin involvement in ZEBOV movement through the cell cytoplasm. Treatment with inhibitors of PI3K, Akt-1 and Rac1 each gave a similar outcome to that seen in the HEK293 cells, with clustering of virus particles within a cytosolic compartment (Figure 6B), possibly of endocytic origin. These observations suggest that in the absence of PI3K activity, virus particles are taken into a vesicular compartment, but further trafficking is blocked.

## Discussion

This study describes a novel role for the PI3K cell signaling pathway in cellular entry of ZEBOV. A number of viruses utilize the PI3K-Akt cell signaling pathway to promote various steps in their replication cycle, such as regulation of gene expression and genome replication. Some bacteria and a few non-enveloped viruses also utilize this pathway to trigger their invasion and endocytosis into cells [22], [29]–[32]. This report provides evidence that the PI3K pathway plays a critical role in cellular entry of ZEBOV. A previous report suggested that PI3K is involved in early events in Influenza virus infection [33]. However, a detailed analysis of the mechanism of action was not performed and a subsequent study using the same cell type and virus strain failed to show a requirement of PI3K activity for Influenza virus entry into cells [34]. Thus, the present report is the first to show involvement of the PI3K-Akt pathway in entry of an enveloped virus.

The data obtained using the knockout cells provided information on which subtype of PI3K was important. Inhibition of pseudotyped ZEBOV entry into pik3R1 knockout (p85α-, p50α-, p55α-deficient) or pik3R2 knock out (p85β-deficient) cells suggested that class IA PI3Ks played a prominent role. Entry inhibition was more pronounced in pik3R2 knockout cells than in the pik3R1 knockout cells. This was somewhat surprising given that p85α is a major PI3K regulatory subunit, and deletion of the pik3R1 gene has a greater phenotypic impact, including perinatal lethality of homozygous mice accompanied with extensive hepatocytic and brown fat necrosis, enlarged skeletal muscle fibers, calcification of cardiac tissue and impaired B-cell development and proliferation [35],[36]. However, a few responses such as T-cell proliferation, and insulin-dependent tyrosine phosphorylation of insulin receptor substrate-2 were increased in pik3R2 knockout mice [37],[38], indicating that each subtype may have specialized roles in specific cells and tissues. Indeed, each binds different sets of proteins [39]. Individual disruption of pik3R1 or pik3R2 gene was insufficient to confer complete inhibition of ZEBOV entry and may be due to a partial redundancy in the function of each or that other PI3K isoforms may also be involved.

Given the relatively slow entry kinetics of ZEBOV, compared to VSV, the rapid phosphorylation of Akt by ZEBOV suggests that induction of the PI3K pathway may be related to a very early event in the entry process, such as receptor/co-receptor engagement. The class 1A PI3Ks are mainly activated by membrane-bound receptor tyrosine kinases (RTKs) [40]. Recently, Axl, Dtk, and Mer (Tyro3 family RTKs) were shown to serve as important entry factors for Ebola and Marburg viruses [41]. It was suggested that these molecules serve to promote endocytosis of ZEBOV particles; however, the exact mechanism and downstream effectors remained unclear. Interestingly, the inhibition of PI3K or Akt caused virus particles to aberrantly accumulate within the cell cytoplasm. In many cell types tyro3 family members trigger PI3K activation, and physical association of PI3K with Axl, Dtk and Mer has also been demonstrated [42]. ZEBOV interaction with the tyro3 RTK may then directly or indirectly trigger activation of PI3K and downstream effectors leading to virus endocytosis. However, more detailed analyses are required to further test this model.

In further studying the mechanism of action, Rac1 was found to be an important downstream effector. PI3K-Akt is one prominent activation pathway for Rac1 in many cell types [13]–[16]. Treatment with PI3K, Akt or Rac1 inhibitors all led to similar intracellular accumulation of ZEBOV particles (Figure 6), signifying a common block in one of the stages of ZEBOV uptake into cells. A similar clustering of internalized EphA2 receptor in endocytic vesicles was also observed after treatment with inhibitors of PI3K, Akt or Rac1 [43]. The likely role of Rac1, was through its regulation of actin polymerization, which plays a pivotal role in a variety of actin-dependent cellular processes such as membrane ruffling, receptor-mediated endocytosis and vesicular trafficking [12]. Indirect evidence suggesting that control of actin polymerization may be important for ZEBOV infection came from observations that: (i) internalized ZEBOV particles were in close proximity to actin bundles or filaments; (ii) inhibitors of PI3K, Akt and Rac1 all caused similar changes in F-actin characterized by loss of membrane ruffling and focal adhesions (data not shown); and (iii) agents that perturb actin dynamics significantly inhibit EVP entry [26].

Early activation of the PI3K-Akt pathway by ZEBOV may also have implications in pathogenesis of Ebola virus hemorrhagic fever. A profound inflammatory response is a key feature of the disease. Macrophages are among the primary targets of ZEBOV infection and respond by producing a number of proinflammatory cytokines and chemokines including TNF-α, IL-6 and IL-8 [44]. This appears to occur in the absence of virus replication as Ebola virus-like particles (VLPs) stimulate the same set of cytokines and is dependent on the presence of the Ebola virus envelope glycoproteins [45]. It follows that Ebola virus envelope proteins may play a vital role in the proinflammatory response induced during the infection. There is also evidence that the PI3K-Akt pathway contributes significantly toward regulation of each of these cytokines [46]–[49]. ZEBOV-induced activation of the PI3K-Akt pathway could then directly contribute to the proinflammatory response. Another hallmark of ZEBOV infection is hemorrhage due to increased vascular permeability. Vascular dysregulation has been attributed to both direct invasion and replication of ZEBOV in vascular endothelial cells, and to action of ZEBOV-induced proinflammatory cytokines, especially TNF-α, on vascular endothelial cells [50]. Also, PI3K-Akt pathway activation does lead to increased vascular permeability [51]. Thus, whether the mechanism of vascular dysregulation is through virus replication or action of cytokines, ZEBOV-induced PI3K activation has ability to affect both mechanisms, and thereby can potentially influence and partly explain a mechanism of ZEBOV pathogenesis.

The PI3K pathway is vital for regulation of diverse cellular activities, including growth, survival, differentiation and motility. There is mounting evidence that aberrant regulation of the PI3K pathway is central to development and/or progression of many forms of cancer [40]. As a result, considerable effort is currently being focused on developing therapeutic strategies targeting various components of this pathway, including targeting specific isoforms and subunits of the PI3K holoenzyme [52]. Earlier this year a PI3K inhibitor entered phase 1 clinical trials indicating that such drugs are becoming available [53]. The finding that the PI3K pathway is also essential for entry of ZEBOV is therefore highly relevant for design of new therapeutic strategies and provides new potential opportunities where PI3K inhibitors developed for cancer treatments may become equally useful for treatment ZEBOV infection.

## Materials and Methods

### Cells and culture

HEK293 and HEK293FT human fibroblast-derived cells were purchased from ATCC and Invitrogen, respectively. HEK293-mCAT-1 are a clonal derivative of HEK293 cells that express the mCAT-1 protein which serves as a receptor for ecotropic murine leukemia viruses (MLV), such as the Friend 57 strain of MLV (Entrez nucleotide #X02794). These cells have similar morphology and growth properties compared to the parental HEK293 cells. Inhibition of ZEBOV entry by PI3K inhibitors was also confirmed in HEK293 cells with similar outcomes (data not shown). Vero-E6 cells were also purchased from ATCC. HEK293, FT and Vero-E6 cells were maintained in Dulbecco's modified Eagle's (DMEM) medium supplemented with 10% fetal bovine serum (Gemini Bioproducts, GA), 1% non-essential amino acids (Sigma, MO) and 1% penicillin-streptomycin solution (Sigma, MO). HEK293FT cells were used for pseudotype production and were maintained in the presence of geneticin at 0.5 mg/ml. Mouse embryonic fibroblasts (MEFs) were isolated from embryos that were heterozygous or homozygous for knockout of the PI3K-family related genes pik3R1 and pik3R2. Immortalized cells from these embryos were provided by Dr. Lew Cantley (Harvard Medical School, MA) and were cultivated in the above medium.

### Antibodies

ZEBOV-specific KZ52 monoclonal antibody was a gift from Dr. Dennis Burton (Scripps Research Institute, LaJolla, CA). Anti-Akt-1 and anti-phospho-Akt-1 antibodies were purchased from Cell Signaling Technologies (Danvers, MA). Anti-HA (12CA5) was used as a non-specific control monoclonal antibody (Roche, IN).

### Plasmid constructs

All plasmids were prepared using Qiagen kits or by CsCl gradient centrifugation following standard procedures. The plasmids encoding HIV-1 gag and polymerase (pLP1), HIV-1 Rev (pLP2) and VSV-G envelope glycoprotein (pLP-VSVG) were purchased from Invitrogen. Construction of the plasmids encoding packageable enhanced green fluorescent protein (pLenti-EGFP) and Nef-luciferase fusion protein (pCDNA3-nef-luc) has been described previously [24]. Plasmid encoding ZEBOV matrix protein (VP40) (Entrez gene#NC002549) and envelope glycoproteins were kindly provided by Dr. Christopher Basler (Mount Sinai School of Medicine) and Dr. Paul Bates (University of Pennsylvania), respectively. The plasmid encoding envelope protein of the Friend 57 strain of MLV (pFr-Env) has been described previously [54].

### Production of pseudotyped viruses containing nef-luciferase fusion protein

HEK293FT cells were grown to approximately 80% confluence in 10-cm diameter dishes. The cells were simultaneously transfected with plasmids: (i) pLP1 (3 µg), pLP2 (2 µg), pLenti-EGFP (2 µg), pcDNA3-Nef-luc (1.5 µg) and one of the following envelope protein constructs pLP-VSVG, 2 µg; pFr-Env, 5 µg; pEbola-GP, 0.5 µg to yield pseudotyped viruses with VSV, Friend 57 MLV or ZEBOV envelope glycoproteins respectively. Transfection was by calcium-phosphate precipitation [55]. After overnight incubation, culture medium was replenished and the plate was incubated for a further 36 h. At this time, the cell culture supernatant was collected and filtered through a 0.45-µm pore size cellulose-acetate filter to remove cell debris. Virus in the filtrate was pelleted by centrifugation through a 20% (w/v) sucrose cushion in PBS. Centrifugation was for 3.5 h at 25,000 rpm in SW28 rotor at 4°C. The virus pellet was resuspended in 0.01 volume of DMEM, aliquoted and stored at −80°C until used.

### Production of virus-like particles containing Nef-luciferase fusion protein

ZEBOV-VLPs were produced by co-transecting HEK293 cells with plasmids encoding ZEBOV matrix protein (VP-40), Zaire Ebola virus (ZEBOV) envelope glycoproteins, and Nef-luciferase fusion protein using the calcium phosphate method. For VSV-VLP, plasmid encoding Ebola virus glycoproteins was replaced with one encoding VSV-G. Cell culture supernatant was collected 48 h after transfection and cell debris was cleared by centrifugation (1,200 rpm for 10 min at 4°C). Subsequently, VLPs were purified by centrifugation (25,000 rpm in SW28 rotor for 3.5 h at 4°C) through a 20% (w/v) sucrose cushion in PBS. The VLP pellet was resuspended in 0.01 volume of DMEM, aliquoted and stored at 4°C. Assays were performed within 2–3 days after purification of VLPs.

### Cultivation of ZEBOV and determination of virus titer

Zaire Ebola virus (Mayinga strain), was cultivated on Vero-E6 cells by infection at an MOI of 0.1. Culture supernatants were collected after 10 d and clarified by centrifugation at 2000×g for 15 min. Virus titer was determined by serial dilution in Vero-E6 cells. Cells were incubated with virus for 1 h and then overlaid with 0.8% (w/v) tragacanth gum in culture medium. 10 d post-infection cells were fixed with formalin, and stained with crystal violet so that plaques could be counted. All experiments with ZEBOV were performed under biosafety level 4 conditions in the Robert E. Shope BSL-4 Laboratory, UTMB.

### Determination of pseudotype virus titer

HEK293-mCAT-1 cells were grown in a 96-well plate to approximately 50% confluence. Serial 5-fold dilutions of virus stocks were prepared in DMEM and 50 µl of each dilution was added to cells. After overnight incubation, the medium was replenished and the incubation continued until GFP expressing cells were apparent (2 d post-infection). The total number of GFP-positive colonies was counted in each well using an inverted epifluorescence microscope and the titer of stock virus was calculated.

### Virus entry assay

Cells were removed from plates by trypsin treatment, pelleted by centrifugation and then resuspended in fresh medium. HEK293 cells (10^6^) were mixed with Nef-luciferase containing pseudotyped virus or VLPs in a volume of 0.2 ml and incubated at 37°C on a rotating platform for indicated time intervals. Exposure of cells to low temperatures (4°C) was avoided as this is known to temporarily disrupt endocytosis and receptor trafficking upon return to 37°C. To remove excess virus particles, cells were pelleted by centrifugation at 200×*g* for 5 min, supernatant containing unbound virus was discarded, and the cell pellet was washed 3 times with DMEM. The final cell pellet was resuspended in 0.1 ml of luciferase assay buffer lacking detergent (Promega, WI) and luciferase activity measured using a Turner Design TD 20/20 luminometer and expressed as counts/sec.

For antibody inhibition assays, the luciferase-containing pseudotyped virus or VLPs were incubated with antibody for 1 h prior to incubation with target cells, which was performed in the continued presence of antibody.

To study drug activity on virus entry, cells were pre-treated for 1 h, followed by incubation with pseudotyped virus or VLPs in the continued presence of the drug. Virus entry was then measured as described above.

For dominant-negative or constitutively-active mutants, control plasmid (pcDNA3) or plasmid encoding the modified cDNA was transfected into HEK293-mCAT-1 cells by calcium phosphate precipitation as described above. Cells were used for entry assays 36 h after transfection.

### Analysis of Akt-1 phosphorylation

HEK293 cells were grown to confluence and then serum-starved for 12–14 h. Radiation-inactivated wild type ZEBOV (Entrez Genome#15507) or VSV (Entrez Genome#10405) (sucrose purified and resuspended in serum-free medium) was then added at a calculated MOI of 5. For positive control, cells were treated with 10% fetal bovine serum in medium, while the negative control samples received serum-free medium. All samples were incubated at 37°C for times indicated. After the incubation, cell lysates were applied to 10% polyacrylamide gels and resolved proteins transferred to a nitrocellulose membrane by electroblotting. After blocking the membrane in 5% milk powder in TBST, blots were incubated overnight with anti-phospho-Akt-1 antibody at 4°C, washed and incubated with HRP-conjugated secondary antibody for 1 h. The membrane was then washed and developed using ECL chemiluminescence substrate (GE life sciences, Piscataway, NJ) and imaged. Subsequently, the same membrane was stripped and re-probed for total Akt-1 using an anti-Akt-1 antibody. Band densitometry was performed using ImageJ analysis software [56].

### Labeling of ZEBOV with fluorescent dye

ZEBOV was grown on Vero-E6 cells to a titer of 10^6^ pfu/ml. Virus-containing culture supernatant was clarified by pelleting cell debris at 2000×g for 15 min. The virus remaining in the supernatant was then pelleted through 20% sucrose in 10 mM HEPES, pH 7.4 by centrifugation at 100,000×g for 3 h. The virus pellet was resuspended in 140 mM NaCl in 10 mM HEPES, pH 7.4 and inactivated by gamma-radiation (5 Mrad). Protein content of the virus pellet was determined using a BCA protein assay kit (Pierce, Rockford, IL). An equal volume of 0.1 M sodium phosphate, pH 8.0 was added and protein concentration adjusted to 2 mg/ml by further addition of this buffer. Of this, 0.1 mg of total protein was labeled with 0.05 mg of Alexa Fluor_594_ carboxylic acid, succinimidyl ester (Invitrogen). The reaction was allowed to proceed for 2 h at room temperature at which time it was quenched by addition of 0.1 volume of 0.1 M glycine. The samples were then dialyzed overnight against PBS at 4°C and then again overnight against DMEM. The virus suspension was then aliquoted and stored at −80°C.

### Microscopy

HEK293-mCAT-1 or Vero-E6 cells were cultivated overnight on chambered coverglass (Nunc, Rochester, NY) at a density of 50%. The following day, cells were incubated with fluorescently-labeled ZEBOV for 3 h. For analysis of drug action, cells were pretreated for 1 h prior to virus addition as described above. Cells were then washed three times in DMEM and fixed in 3.5% fresh paraformaldehyde in PBS. After one wash in PBS residual paraformaldehyde was neutralized by addition of 0.1 M glycine buffer, pH 7.4 and cells were permeabilized using 0.1% Triton X-100 for 1 min at room temperature. Cells were stained for F-actin using Alexa_488_-conjugated phalloidin (Invitrogen) for 15 min at room temperature. Cells were imaged using a Leica DMIRB inverted microscope with a 100× oil immersion lens or a Zeiss LSM 510 confocal microscope in the UTMB optical imaging core.
